# Multi-omic analysis reveals HIP-55-dependent regulation of cytokines release

**DOI:** 10.1042/BSR20200298

**Published:** 2020-03-20

**Authors:** Yunqi Jiang, Zihao Xing, Baolin Zhu, Wenjing Wang, Yang Sun, Zhi Shi, Zijian Li

**Affiliations:** 1 Department of Cardiology and Institute of Vascular Medicine, Peking University Third Hospital, Key Laboratory of Cardiovascular Molecular Biology and Regulatory Peptides, Ministry of Health, Key Laboratory of Molecular Cardiovascular Sciences, Ministry of Education, Beijing Key Laboratory of Cardiovascular Receptors Research, Beijing 100191, China; 2 Department of Cell Biology and Institute of Biomedicine, National Engineering Research Center of Genetic Medicine, Guangdong Provincial Key Laboratory of Bioengineering Medicine, College of Life Science and Technology, Jinan University, Guangzhou, Guangdong 510632, China

**Keywords:** Bioinformatics, Cytokine, HIP-55, IL-6, Microarray

## Abstract

HIP-55 (HPK1 [hematopoietic progenitor kinase 1] -interacting protein of 55 kDa) contains an actin-depolymerizing factor homology (ADF-H) domain at the N-terminus and a src homology 3 (SH3) domain at the C-terminus, which plays an important role in the T cell receptor (TCR) and B-cell receptor (BCR) signaling and immune system. In our previous studies, HIP-55 was found to be highly expressed in several types of tumors and function as a novel oncogenic signaling hub that regulates tumor progression and metastasis through defined functional domains, actin-binding and SH3 modules. However, the wider functions and mechanisms of HIP-55 are still unclear. Here, multi-omic analysis revealed that one of the main biofunctions of HIP-55 is the regulation of cytokines release. Furthermore, to investigate the role of HIP-55 in the cytokine production, a series Cytokine Antibody Arrays were performed to detect differentially expressed cytokines between control and HIP-55 knockdown cells. A total of 97 differentially expressed cytokines were identified from 300 cytokines in A549 cell. Bioinformatics analysis showed these differentially cytokines were mainly enriched in cancer signal pathways and IL-6 is the most critical hub in the integrated network. Analysis of RNAseq data from lung cancer patients showed that there is a strong negative correlation between HIP-55 and interleukin-6 (IL-6) in samples from lung adenocarcinoma patients. Our data indicated that HIP-55 may participate in cancer progression and metastasis via regulating cytokines release.

## Introduction

Cancer is one of the leading causes of death in worldwide for its extreme complex pathogenesis and rapid progression, and its incidence is still increasing in recent years. Though progress in early diagnostic biomarkers and precise treatments have been achieved, the average 5-year survival rates for malignant is still low [1]. New therapy targets for malignancy are needed urgently.

HIP-55, hematopoietic progenitor kinase 1 (HPK1)-interacting protein of 55 kDa, also called mabp1(mammalian homologue of the yeast Abp1), which contains an actin-depolymerizing factor homology (ADF-H) domain at the N-terminus and a src homology 3 (SH3) domain at the C-terminus [2,3]. Our previous studies have demonstrated that HIP-55 were over-expressed in several types of tumor tissues, such as thyroid cancer and lung cancer [4,5]. Furthermore, HIP-55 showed significant promotive effects in the proliferative and invasive activity of lung cancer cells and the apoptosis-inhibiting effects on A549 cells [4,5]. Thus, our accumulative evidence suggested that HIP-55 may be important for tumorigenesis and progression. However, the detailed mechanisms of HIP-55 participated in cancer were still unclear.

Microarray analyses for the mRNA expression profiling of cancer cell lines have revealed many specific biological gene patterns in various cancers. The gene-expression signatures of mRNA of cancer cells also have been utilized in improving diagnostic accuracy, predicting the prognosis and providing information for precise therapy [6]. In the present study, we performed a high-throughout method to definite the biofunctions of HIP-55. We presented an mRNA profile analysis in both HIP-55 knockdown (KD) A549 cells and control A549 cells. The bioinformatics analysis demonstrated these differentially expressed genes (DEGs) were mainly involved in cytokine production. Then, we utilized a series cytokine arrays to examine the cytokine expression profiles in HIP-55 KD cells. Bioinformatics analysis exhibited that the changed cytokines were mainly enriched in cancers signaling pathways and interleukin-6 (IL-6) was located in the core of the integrated network. In specimens from lung adenocarcinoma patients, correlation analysis of RNAseq revealed that there was a negative correlation between HIP-55 and IL-6. Our results demonstrated a possible mechanism of HIP-55 participated in cancer progression and may provide a new therapy target for cancer.

## Materials and methods

### Cell culture and transfections

The lung adenocarcinoma cell line A549 was cultured in Dulbecco’s modified Eagle’s medium with 10% fetal bovine serum, incubated at 37°C in humidified air with 5% CO_2_.

### Stable HIP-55 knockdown cell lines generation

The gene sequence of HIP-55 was designed as AACAGTGAACGTAGAGAATTG. To generate stable HIP-55 knockdown cell lines, A549 cells were infected by recombinant retroviruses which carrying HIP-55 shRNA. Then, the cells were selected by applying puromycin. Drug-resistant clones were collected, pooled and expanded. The HIP-55 level was verified for every experiment.

### Real-time PCR analysis

Total RNA was extracted from A549 cells by using the TRIzol reagent. About 1 µg RNA was reverse transcribed into cDNA using the ImProm-II reverse transcription system (Promega, WI, U.S.A.). Real-time PCR was performed using the GoTaq® 2-Step RT-qPCR System for Dye-Based Detection System (Promega, WI, U.S.A.). Relative mRNA levels were quantified using the comparative ∆*C*_T_ method, and then normalized to beta actin. The following primer were used for RT-PCR: TNFSF9: 5′-GCGGTGCAATCATGAGTCAA-3′ (forward) and 5′-ACACAACTGTGGTCCCAGCTACT-3′ (reverse); SOX2: 5′-TAAGTACTGGCGAACCATCTCTGT-3′ (forward) and 5′-TTGGGATCGAACAAAAGCTATTATAA-3′ (reverse); MMP7: 5′-CCTGTATGCTGCAACTCATGAACT-3′ (forward) and 5′-TGGATACATCACTGCATTAGGATCA-3′ (reverse); CDH1: 5′-GCCTGCTTTTGATGATGTCTACA-3′ (forward) and 5′-TTCTGTGCACACCTGGAATTG-3′ (reverse); IL-6: 5′-CTGCGCAGCTTTAAGGAGTTC-3′ (forward) and 5′-TGAGGTGCCCATGCTACATTT-3′ (reverse); Beta actin: 5′-CTGGAACGGTGAAGGTGACA-3′ (forward) and 5′-CGGCCACATTGTGAACTTTG-3′ (reverse).

### Gene expression analysis

Gene expression analysis was carried out using Affymetrix GeneChip Human Genome U133 Plus 2.0 expression arrays and following the instructions to get the raw data. Raw data were normalized by MAS 5.0 algorithm. Different expressed genes were defined as fold change ≥2 or ≤0.5.

### Bioinformatics analysis

The Bioinformatics data were obtained from the omics bean software (www.omicsbean.cn), a web-delivered application that analyze the biological process analysis, GO (gene ontology) enrichment analysis, the KEGG pathway enrichment analysis, etc.

### Western blot

Equivalent proteins were separated by SDS-PAGE, then transferred to the PVDF membranes, and blocked by TBST containing 5% skim milk for an hour at room temperature, and then, PVDF membranes were incubated at 4°C overnight with primary antibodies (HIP-55, sc-398498, 1:1000; beta-actin, sc-517582, 1:1000), and then incubated with secondary antibodies for an hour at room temperature. Finally, the membranes were visualised with the enhanced chemiluminescence (Millipore, MA, U.S.A.) in the dark room.

### Cytokine antibody array

Cytokines were detected in cell conditioned medium with human antibody array analysis [RayBio® Cytokine Antibody Arrays G series 6 -10] (RayBiotech Inc, GA, U.S.A.), following the manufacturer's instructions, a total of 300 cytokines were simultaneously screened. The chips were scanned using a laser scanner and saved digitally, and the optical density was measured using ImageJ software (NIH, MD, U.S.A.). The relative expression of cytokines was calculated by using the intensities of the corresponding protein fluorescence after background correction and normalization.

### IL-6 ELISA

Cells released IL-6 levels were measured by human IL-6 ELISA (Abcam, Cambridge, U.K.), according to the manufacturer’s instructions. Measurements of density (OD) at 405 nm were recorded.

### Analysis of lung adenocarcinoma RNAseq data

RNA sequence data for 515 lung adenocarcinoma (LUAD) samples and 59 normal lung samples and clinical xml data were downloaded from the The Cancer Genome Atlas (TCGA) Launch Data Portol (https://portal.gdc.cancer.gov). Then the HIP-55 and IL-6 data sets were extracted from the whole RNAseq count data and FPKM data. The expression and correlation of HIP-55 and IL-6 in different tissues were analyzed by DEseq2 package of R [7].

### Statistical analysis

Student’s *t* test was used to compare individual data between each group. Downloaded RNAseq data were evaluated by Spearman correlation and linear regression analysis by using the R statistical programming environment. *P* < 0.05 was considered as a statistically significant difference.

## Results

### Bioinformatics analysis of the differentially expressed genes associated with down-regulation of HIP-55

**Figure 1:**
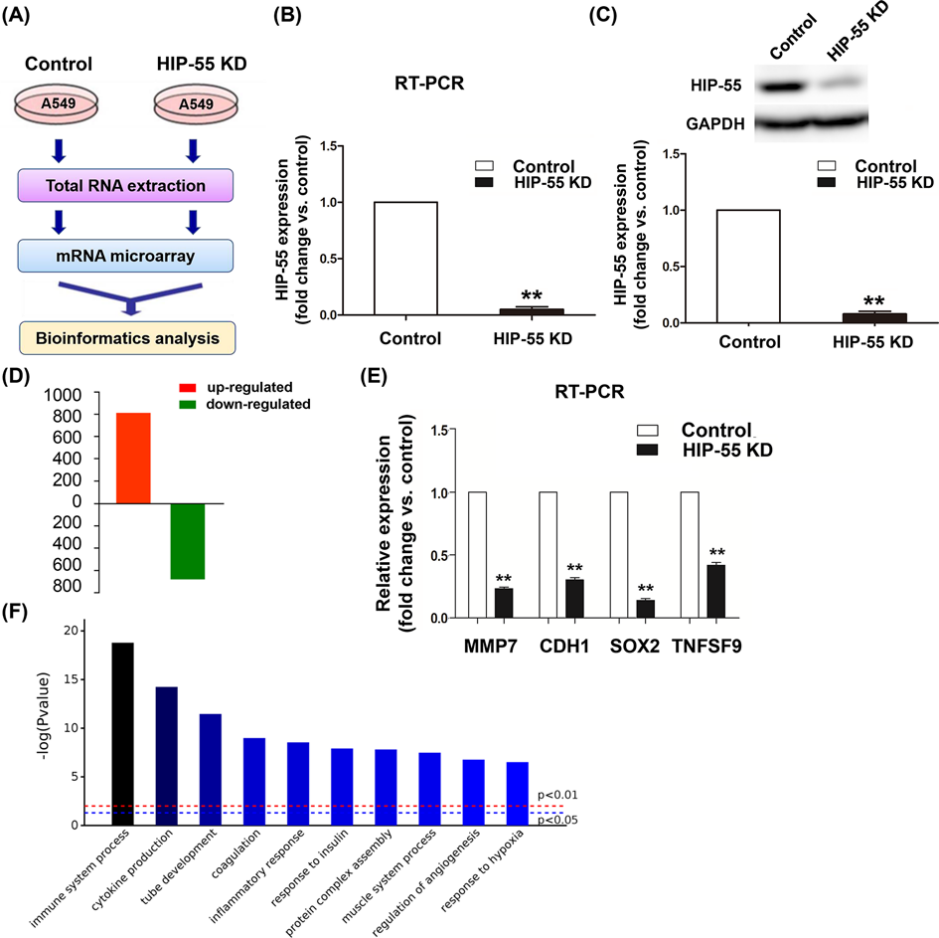
Bioinformatics analysis of the differentially expressed genes associated with down-regulation of HIP-55 (**A**) Workflow for bioinformatics analysis of the differentially expressed genes between HIP-55 knockdown A549 cells and control A549 cells. (**B** and **C**) RT-PCR and Western blot were used to detect the mRNA and protein expression of HIP-55. (**D**) Total of 1618 different genes within 847 up-regulated and 771 down-regulated were identified (fold change ≥2 or ≤0.5) in the HIP-55 knockdown group compared with control group. (**E**) Some differentially expressed genes from microarray data were validated by RT-PCR. (**F**) Biological process analysis of the differentially expressed genes. vs control, **P ≤ 0.01.

In order to study the biological function of HIP-55 and to identify additional protein targets for HIP-55 in A549 cells, mRNA microarray was applied to in both HIP-55 knockdown cells and the control cells (Figure 1A). The stable HIP-55 knockdown A549 cell line was established with recombinant retroviruses carrying HIP-55 shRNA. Compared to control cells, the expression of HIP-55 in both mRNA and protein levels was significantly decreased in the HIP-55 knockdown cells with effective knockdown rate >90% (Figure 1B,C).

Compared with the control group, a total of 1618 DEGs were identified as fold change ≥2 or ≤0.5, of these, 847 DEGs were robustly up-regulated and 771 DEGs were down-regulated (Figure 1D) associated with down-regulation of HIP-55.

To validate the results of mRNA microarray analysis, RT-PCR analysis was performed to evaluate some DEGs mRNA levels, including MMP7, CDH1, SOX2 and TNFSF9. As expected, the results were consistent with the results of microarray assay (Figure 1E).

Furthermore, the differentially expressed genes underwent bioinformatics analysis to investigate the critical relevant biological process. As shown in Figure 1F, the top two terms were immune response and cytokine production. Previous studies mostly focused on the effects of HIP-55 on regulation of immune system, however, little research have been done to explore the regulation of cytokine production.

### Identification of differentially expressed cytokines associated with HIP-55

**Figure 2:**
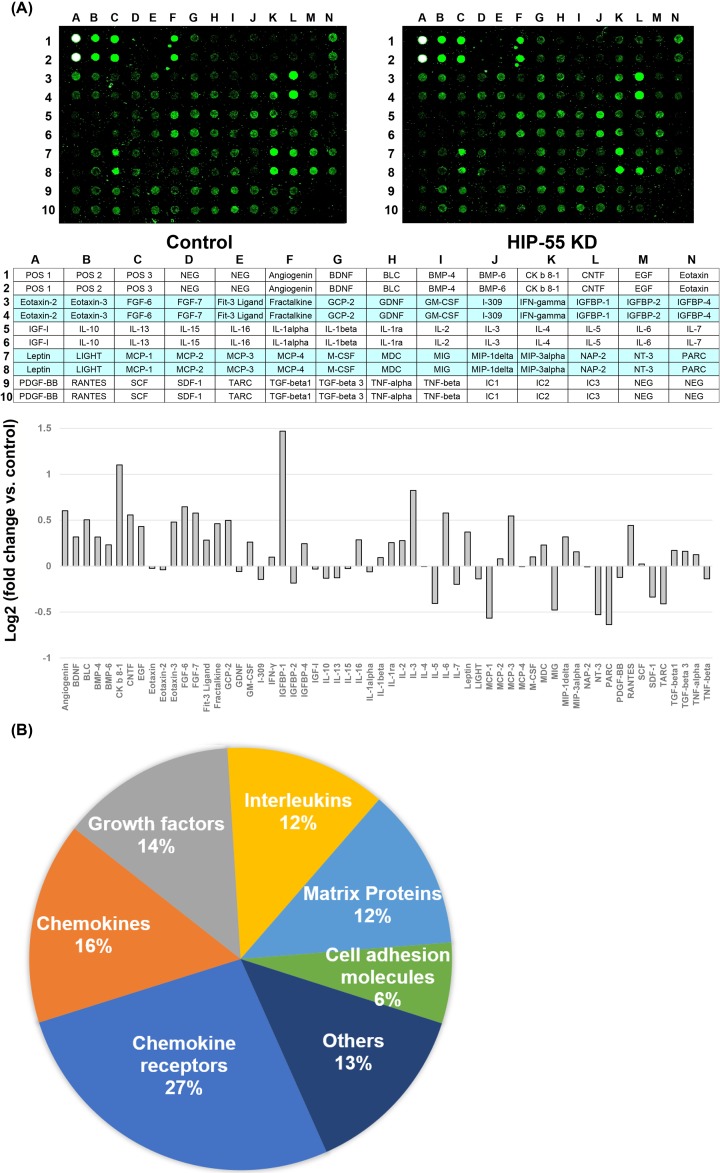
Profiling of cytokines expression in control and HIP-55 knockdown A549 cells RayBio® Human Cytokine Antibody Array System G Series 6-10 were performed to detect 300 cytokines levels in both control and HIP-55 knockdown A549 cells. (**A**) Representative fluorescent signal images for Human Cytokine Antibody Array G6. Upper panel shows representative blots of 60 cytokines. The middle panel shows the place of each cytokine within the Human Cytokine Antibody Array System G 6 and the lower panel shows the relative expression of each cytokine within the Human Cytokine Antibody Array System G6 in HIP-55 KD group compared with control group. (**B**) Total of 97 cytokines changed, chemokine receptors made up 27%, followed by chemokines (16%), growth factors (14%), interleukins (12%), matrix proteins (12%) and cell adhesion molecules (6%) and others (13%).

In order to further study how HIP-55 affects cytokine production, we performed a series cytokine antibody arrays analysis in both control and HIP-55 knockdown group. The Figure 2A was the representative antibody array and demonstrates the protein spot and signal strength of the proteins. Of these 300 cytokines, 97 cytokines expression levels have changed (Supplementary Table S1), and the major changed proteins were chemokine receptors (27%), followed by chemokines (16%), growth factors (14%), interleukins (12%) and matrix proteins (12%) (Figure 2B).

### Bioinformatics analysis of differentially expressed cytokines

**Figure 3:**
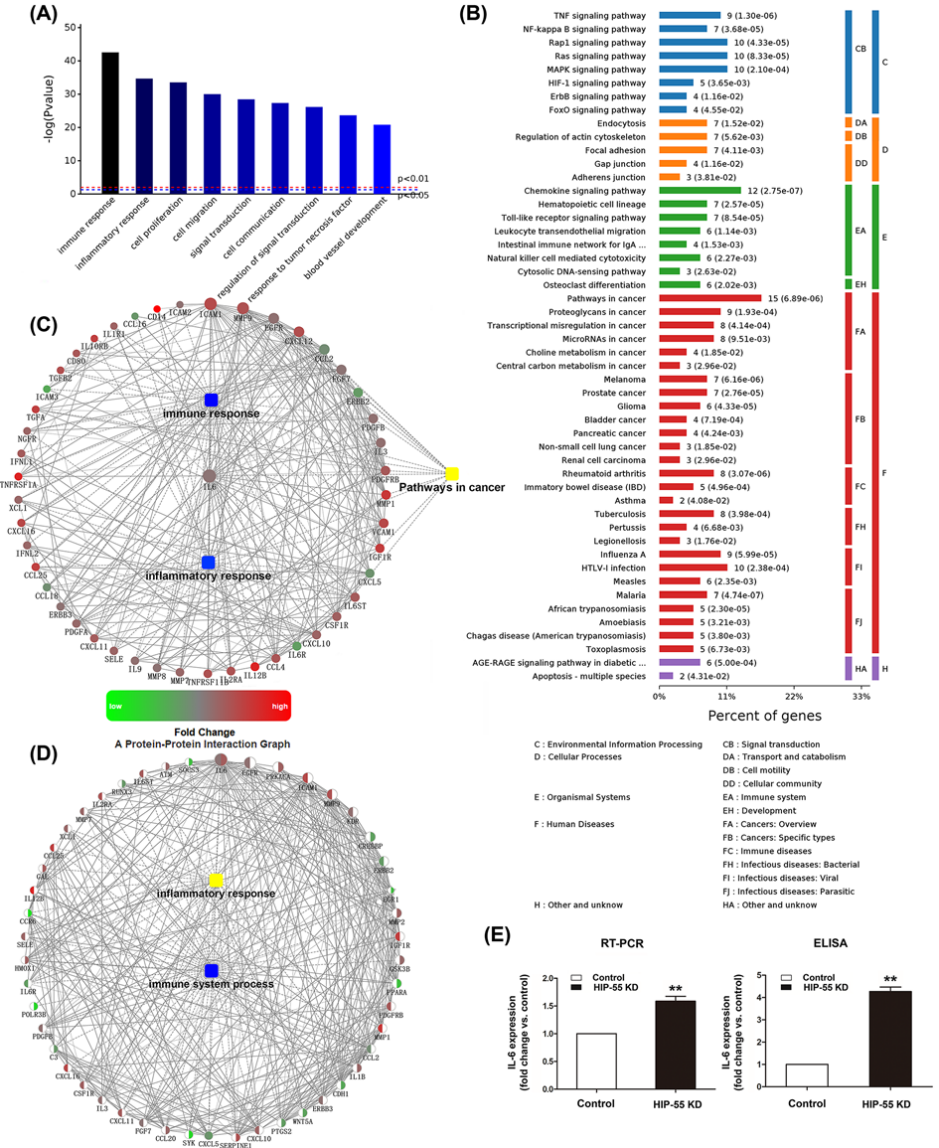
Bioinformatics analysis of different expressed cytokines associated with down-regulation of HIP-55 (**A**) Biological process analysis of the differentially expressed cytokines. (**B**) The KEGG pathway enrichment analysis reveals that these changed cytokines were enriched in several pathways. (**C**) In the upper panel, protein- protein interaction network was performed which containing immune response, inflammatory and pathways in cancer, and IL-6 is at the core of the network. The meaning of the different colors and shades was indicated in the lower panel. (**D**) Integrated protein-protein interaction analysis of different expressed mRNA and cytokines. (**E**) Detection of IL-6 transcription and translation levels by RT-PCR and ELISA in HIP-55 KD group. vs control, **P ≤ 0.01.

Bioinformatics analysis was performed to analyze the influence on cellular biological processes of the differentially expressed cytokines. Results revealed that these cytokines were mainly involved in immunoreaction, inflammation, cell proliferation and other pathophysiologic processes (Figure 3A). Based on the KEGG pathway analysis, these cytokines were enriched in some pathways including TNF signaling, NF-κB signaling pathway, chemokine signaling pathway and some other pathways. Nevertheless, compared with the other signaling pathway, many cytokines were significantly enriched in cancer signal pathway (Figure 3B).

As shown above, the differentially expressed cytokines were predominantly involved in the immune response and the inflammatory response biological process. As well known, immune and inflammation were the core process of the tumor microenvironment, one of the ten hallmarks of cancer [8]. Therefore, we performed an protein–protein interaction network containing immune response, inflammatory and pathways in cancer. In the complex network, IL-6 played a central role and was associated with multiple modules, indicating that IL-6 would be important in regulating the expression of numerous genes and connecting various pathways (Figure 3C). Since the mRNA microarray reveals the intracellular changes while cytokine array uncovers the extracellular changes, we performed an integrated protein–protein interaction analysis of different expressed mRNA and cytokines. Results showed that these different expressed genes and cytokines were enriched in inflammatory and immune response as well (Figure 3D).

As an extensively studied multifunctional cytokine, IL-6 and its receptor IL-6R/gp130 complex, has been reported to be associated with various tumors for its role in inflammation, immunoreaction, cellular survival and so on [9]. Since the bioinformatics gave a hint that HIP-55 is closely related to IL-6, we wanted to identify the definite correlation between HIP-55 and IL-6 in A549 cells. We performed RT-PCR and ELISA to detect IL-6 levels and results showed that IL-6 expression was decreased at both the mRNA and protein levels in HIP-55 KD group (Figure 3E). Thus, our results indicated a robust negative correlation between HIP-55 and IL-6 at gene transcription and translation levels.

### Expression and correlation analysis of HIP-55 and IL-6 in lung adenocarcinoma patients

**Figure 4:**
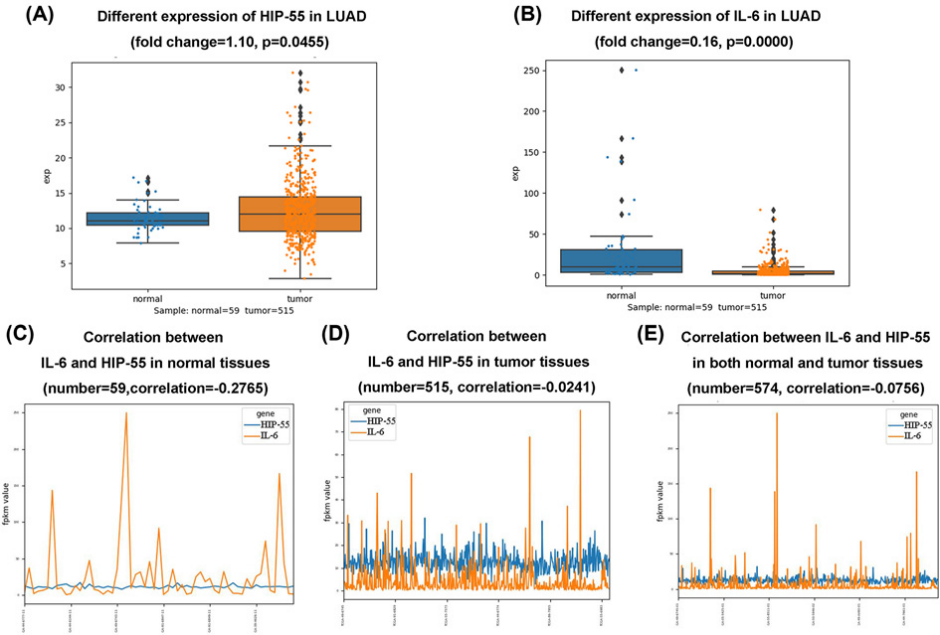
The expression and correlation analysis of HIP-55 and IL-6 in lung adenocarcinoma samples By using TCGA database, 515 tumor tissues and 59 normal tissues were analyzed. The HIP-55 level was higher in tumor tissues than that in normal tissues (fold change = 1.10, *P* = 0.0455) (**A**) and the IL-6 level in the tumor tissues was lower than that in the normal tissues (fold change = 0.16, *P* = 0.0000) (**B**). Correlation analysis showed that HIP-55 had a positive correlation with IL-6 in the normal tissues (*n* = 59, correlation = -0.2765) (**C**) and in the tumor tissues (*n* = 515, correlation = -0.241). (**D** and **E**) HIP-55 had a negative correlation with IL-6 in both the normal tissues and tumor tissues (*n* = 574, correlation = -0.075).

The Cancer Genome Atlas (TCGA) database deepened our understanding of cancer biology, improved our ability to diagnose, treat, and prevent cancer [10,11]. To identify the definite correlation between HIP-55 and IL-6 in lung adenocarcinoma patients, we conducted an expression and correlation analysis of RNAseq between HIP-55 and IL-6. We examined RNAseq data of 574 samples which containing 515 cancer samples and 59 normal samples from TCGA database. The expression analysis indicated a significantly higher transcriptional level of HIP-55 in tumor tissues compared with normal tissues (*P* < 0.01) (Figure 4A), and IL-6 was lower expression in tumor tissue compared to normal tissue (Figure 4B). Correlation analysis showed that there was a strong negative correlation between HIP-55 and IL-6 in lung adenocarcinoma patients (Figure 4C–E).

## Discussion

As a classification of signal molecules, cytokines play an important role in normal physiology activity and its imbalance would leading to numerous diseases (infection, metabolic syndrome, malignant tumors, etc.) [12,13]. Among them, cancer is the most serious disease that threaten human being health. Formation and progression of tumor are very complex multistep processes, in which cytokines occupy critical roles in different stages. Inflammatory factors such as TNF-α could stimulate accumulation of reactive oxygen species and then inducing DNA damage and genomic instability and might lead to initiation of cancer [14]. In the fast-growing period, most solid tumors were hypoxic, these hypoxic tumor cells and some immune cells could secret pro-angiogenesis cytokines such as vascular endothelial growth factor A and growth factors, leading to tumor cell proliferation. In the advanced malignant tumor, chemokines such as CCL5 and CCL18 could enhance adhesion and mobility of cancer cells to accelerate tumor invasion and metastasis [15,16]. Furthermore, some cytokine-targeted drugs have been found to reduce tumor incidence as well as delay cancer progression [14]. As a widely studied cytokine, IL-6 has pleiotropic and complex functions in tumor development. In most cases, IL-6 was high-expressed in cancer and associated with the progression of cancer, resistance to antitumor chemotherapy, and poor prognosis [17]. Once binding with its receptor, IL-6 activates various downstream pathways to regulating tumor formation and progression. However, recent studies have shown that IL-6 may play contrasting biological effects on tumor initiation depending on the types of tumor. IL-6 facilitates osteosarcoma and liver tumor progression, while it inhibits lung and breast cancer development [17,18]. In line with the role of IL6 in lung tumor, the signal transducers and activators of transcription 3 (STAT3), the well defined downstream effector of IL-6, also prevents lung cancer initiation [19].

Complex biofunctions of proteins correspond to their precise spatio-temporal regulation, which also applies to cytokines. Transcriptional regulation is the most fundamental in regulating genes biological function. Several types of transcriptional factor family are considered to play primary roles in controlling the expression of cytokines, such as NF-κB family and NFAT family [20,21]. Regulation of post-translation is also a key for determining expression of cytokines. Among them, microRNA have been considered as a key player in regulating genes expression by forming imperfect 3′ untranslated region of genes to repress translation or induce mRNA degradation. For example, miR-155 deficient mice exhibited enhanced a wide spectrum of interleukins production [22]. Most cytokines are paracrine factors that should be secreted to extracellular to perform biological functions. A highly orchestrated vesicle trafficking is essential for appropriate secretion of cytokines and is precise regulated by some vesicle related proteins [23]. In addition, post-transcriptional mechanisms, such as degradation, are also important to precisely control the production of cytokines [24]. After secretion, most cytokines activate several different signal transduction pathways by binding with its receptors. Upon recognition of cytokines by specific receptors, the cell initiates various signaling pathways to promote numerous biological processes, such as proliferation, migration, differentiation, et al. In the classical cytokine signal pathways, cytokine-receptor complex activates Janus kinases (JAKs) and then JAKs activate STAT transcription factors to regulate genes expression and modulate cellular biological process. Besides JAK/STAT pathways, several cytokines also activate the Mitogen-activated protein kinase (MAPKs) and AKT pathways, as well as some other signal pathways [25]. Thus, those which regulate chemokine receptor activation and/or the corresponding downstream signal transduction could regulate cytokines biofunctions to a great extent as well.

Here, we found HIP-55 regulated many cytokines expression by using a series cytokine arrays. As a signaling adaptor protein containing multi-functional domain, HIP-55 may regulates cytokines biological function in many respects. Previous studies have demonstrated that HIP-55 is important for T-cell receptor (TCR) and B-cell receptor (BCR) signaling transduction, which are important for cytokine production. By regulating cytoskeleton rearrangement though its ADF domain, HIP-55 can bridge the TCR and actin cytoskeleton and further regulate TCR trafficking and endocytosis to modulate TCR signaling transduction [26]. And then negatively regulates the activity of nuclear factor of activated T cells (NFAT) to regulate cytokines production at the transcription level [27]. HIP-55 can inhibit BCR signaling transduction by facilitating BCR endocytosis as well. By regulating actin remodeling, HIP-55 also can recruit inhibitory signaling molecules (such as SH2-containing inositol 5-phosphatase, SHIP-1) to BCR microclusters to negative regulate BCR pathway [28].We also observed that the changes of IL-6 mRNA is not as significant as IL-6 protein level in HIP-55 KD cells (Figure 3E). Previous study has demonstrated that lysosomal degradation is important for modulating cytokine production [24]. By regulating cytoskeleton rearrangement, HIP-55 may also modulate cytokines to deliver to lysosome [29]. Indeed, our unpublished data indicated that HIP-55 can co-localize with lysosomes. Thus, it is likely that HIP-55 may also modulate the lysosomal degradation of IL-6 to regulate IL-6 production.

Though some studies have shown HIP-55 was highly expressed in some different cancers, but less researches have been done to investigate the mechanism of HIP-55 participate in tumor progression [4,5]. Here, we identified that HIP-55 regulated many kinds of cytokines release, such as chemokine receptors, chemokines and other cytokines, and these cytokines were mainly involved in cancer signal pathways. These observations we presented here suggested that HIP-55 may participate in tumor progression and metastasis via regulating cytokines expression. More important, the study also offered a potential new therapy for the malignant tumor.

## Supplementary Material

Supplementary Table S1

## Abbreviations

ADF-H: actin-depolymerizing factor homology
BCR: B-cell receptor
HIP-55: HPK1-hematopoietic progenitor kinase 1
HPK1: hematopoietic progenitor kinase 1
IL-6: interleukin-6
SH3: src homology 3
TCR: T-cell receptor
