# Supplementary material for: Multi-omic analysis reveals HIP-55-dependent regulation of cytokines release
Source: Biosci Rep. 2020 Mar 20;40(3):BSR20200298. doi: 10.1042/BSR20200298 (PMC7087322; doi:10.1042/BSR20200298)
Supplement: Supplementary Table S1 [file BSR-2020-0298_supp.pdf]

Supplementary Table 1

Differentially expressed cytokines associated with down-regulation of HIP-55.

List of 97 cytokines that were found significantly modulated in HIP-55 knockdown A549 cells relative to control cells by the RayBio® Human Cytokine Antibody Array G Series 6-10 Biotin Label-based Antibody Array.

Cytokine category	Gene symbol	Official Name	Fold change (KD/Control)	Classification
Human Cytokine Array G6	ANG	angiogenin	1.51934	Growth factor
	CCL23	C-C motif Chemokine ligand 23	2.14444	Chemokine
	FGF7	fibroblast growth factor 7	1.49205	Growth factor
	IGFBP1	insulin like growth factor binding protein 1	2.77004	Growth factor
	IL3	interleukin 3	1.76851	Interleukin
	IL6	interleukin 6	1.49297	Interleukin
	CCL2	C-C motif Chemokine ligand 2	0.6752	Chemokine
	PARC	C-C motif Chemokine ligand 18	0.64344	Chemokine
Human Cytokine Array G7	AXL	AXL receptor tyrosine kinase	5.96286	Others
	FGF2	fibroblast growth factor 2	2.38511	Chemokine
	EGFR	epidermal growth factor receptor	1.66195	Cytokine receptor
	CXCL5	C-X-C motif Chemokine ligand 5	0.55694	Chemokine
	GRO	(C-X-C motif) ligand 1	0.52798	Chemokine
	CCL16	C-C motif Chemokine ligand 16	0.49498	Chemokine
	ICAM1	intercellular adhesion molecule 1	5.71498	Cell adhesion molecule
	ICAM3	intercellular adhesion molecule 3	0.26679	Cell adhesion molecule
	IGFBP6	insulin like growth factor binding protein 6	27.8291	Growth factor
	IGF1R	insulin like growth factor 1 receptor	8.84156	Cytokine receptor
	Il1r1	interleukin 1 receptor, type I	2.67465	Cytokine receptor
	Il12B	interleukin 12B	0.33679	Interleukin
	Il12B	interleukin 12B	19.8433	Interleukin
	IL6R	interleukin 6 receptor	0.37358	Cytokine receptor
	CXCL11	C-X-C motif Chemokine ligand 11	3.23746	Chemokine
	XCL1	X-C motif Chemokine ligand 1	2.275447	Chemokine
	CCL4	C-C motif Chemokine ligand 16	3.96366	Chemokine
	TNFRSF11B	TNF receptor superfamily member 11b	4.52871	Cytokine receptor
	IL6ST	interleukin 6 signal transducer	3.38913	Interleukin
	TNFRSF1A	TNF receptor superfamily member 1A	21.5401	Cytokine receptor
	CCL25	C-C motif Chemokine ligand 25	8.17541	Chemokine
	TIMP2	Tissue inhibitor of metalloproteinase 4	2.66812	Matrix Protein

	TNFRSF10C	TNF receptor superfamily member 10C	2.11172	Cytokine receptor
Human Cytokine Array G8	CD80	CD80 molecule	3.8362	Others
	BMP7	Bone morphogenetic protein 7	2.96918	Growth factor
	CTF1	Cardiotrophin 1	1.72745	Others
	CD14	CD14 molecule	63.5989	Others
	CXCL16	C-X-C motif Chemokine ligand 16	6.35384	Chemokine
	ENG	endoglin	1.59809	Matrix Protein
	ErbB3	erb-b2 receptor tyrosine kinase 3	1.59128	Cytokine receptor
	SELE	selectin E	2.42939	Others
	ICAM2	intercellular adhesion molecule 1	1.6289	Cell adhesion molecule
	PPDLIM7	PDZ and LIM domain 7	0.65671	Others
	IL10RB	interleukin 10 receptor subunit beta	8.19058	Cytokine receptor
	IL13RA2	interleukin 13 receptor subunit alpha 2	2.77065	Cytokine receptor
	IL18BP	interleukin 18 binding protein	1.60651	Interleukin
	Il18rap	interleukin 18 receptor accessory protein	18.359	Cytokine receptor
	IL2RA	interleukin 2 receptor subunit alpha	4.37745	Cytokine receptor
	IL9	interleukin 9	1.70442	Interleukin
	CXCL10	C-X-C motif Chemokine ligand 10	2.49204	Chemokine
	LAP	like-AP180	2.12236	Others
	LEPR	leptin receptor	1.50926	Cytokine receptor
	CSF1R	colony stimulating factor 1 receptor	2.84641	Cytokine receptor
	MMP1	matrix metallopeptidase 1	10.8688	Matrix Protein
	MMP13	matrix metallopeptidase 13	3.432	Matrix Protein
	MMP9	matrix metallopeptidase 9	4.49684	Matrix Protein
	CCL23	C-C motif Chemokine ligand 23	2.74679	Chemokine
	Ngfr	nerve growth factor receptor	2.39667	Cytokine receptor
	PDGFA	platelet derived growth factor alpha	2.15	Growth factor
	PDGFB	platelet derived growth factor beta	1.66241	Growth factor
	PDGFRB	platelet derived growth factor receptor beta	3.5309	Cytokine receptor
	Pecam1	platelet/endothelial cell adhesion molecule 1	6.1822	Cell adhesion molecules
	CXCL12	C-X-C motif Chemokine ligand 12	5.0021	Chemokine
	TGFA	transforming growth factor alpha	8.06229	Growth factor
	TGFB1	transforming growth factor beta 1	5.0021	Growth factor
	Tie1	tyrosine kinase with immunoglobulin-like and EGF-like domains 1	2.1873	Growth factor
	TIMP4	Tissue inhibitor of metalloproteinase 4	2.14626	Matrix Protein
	CDH5	cadherin 5	2.20891	Matrix Protein
	KDR	kinase insert domain receptor	1.74061	Cytokine receptor

	Fcgr2b	Fc fragment of IgG receptor IIb	1.90471	Cytokine receptor
Human Cytokine Array G9	FURIN	furin	3.13634	Others
	LGALS7	galectin 7	1.4125	Matrix Protein
	GDF15	growth differentiation factor 15	7.88758	Growth factor
	GH1	growth hormone 1	1.50913	Growth factor
	IL10RA	interleukin 10 receptor subunit alpha	1.99052	Cytokine receptor
	IFNL2	interferon lambda 2	2.30451	Others
	IL29	interleukin 29	3.33168	Interleukin
	SCARB2	scavenger receptor class B member 2	1.66519	Cytokine receptor
	MICA	MHC class I polypeptide-related sequence A	3.08564	Others
	MICB	MHC class I polypeptide-related sequence B	66.2084	Others
	MMP7	matrix metallopeptidase 7	2.27301	Matrix Protein
	MMP8	matrix metallopeptidase 8	1.53482	Matrix Protein
	NID1	nidogen 1	3.56554	Matrix Protein
	NRCAM	neuronal cell adhesion molecule	1.97142	Cell adhesion molecule
	SIGLEC9	sialic acid binding Ig like lectin 9	2.23086	Others
	Tace	tumor necrosis factor–α (TNF-α)–converting enzyme	18.3706	Others
	HAVCR1	hepatitis A virus cellular receptor 1	1.85617	Cytokine receptor
	TNFRSF10B	TNF receptor superfamily member 10B	15.3551	Cytokine receptor
	Tsh	thyroid stimulating hormone	4.94374	Growth factor
	TSLP	thymic stromal lymphopoietin	5.42061	Interleukin
	VCAM1	vascular cell adhesion molecule 1	6.24344	Cell adhesion molecule
	TNFRSF9	TNF receptor superfamily member 9	0.19712	Cytokine receptor
Human Cytokine Array G10	Shg	Shotgun	1.76673	Matrix Protein
	PROK1	prokineticin 1	6.24048	Growth factor
	ErbB2	erb-b2 receptor tyrosine kinase 2	0.41638	Cytokine receptor
	IL13RA1	interleukin 13 receptor subunit alpha 1	2.34401	Cytokine receptor
	IL17C	interleukin 17C	0.01585	Interleukin
	IL17F	interleukin 17F	0.02137	Interleukin
	IL17RA	interleukin 17 receptor subunit alpha	0.19079	Cytokine receptor
